# Association of Dry Period Length with Automatic Milking System, Mastitis, and Reproductive Indicators in Cows

**DOI:** 10.3390/ani14142065

**Published:** 2024-07-14

**Authors:** Vigilijus Jukna, Edita Meškinytė, Ramūnas Antanaitis, Vida Juozaitienė

**Affiliations:** 1 Agriculture Academy, Vytautas Magnus University, Universiteto St. 10A, Akademija, LT-53361 Kaunas, Lithuania; vigilijus.jukna@gmail.com (V.J.); edita.meskinyte@vdu.lt (E.M.); 2 Large Animal Clinic, Veterinary Academy, Lithuanian University of Health Sciences, Tilžės Str. 18, LT-47181 Kaunas, Lithuania; 3 Research Institute of Natural and Technological Sciences, Vytautas Magnus University, K. Donelaičio 58, LT-44248 Kaunas, Lithuania

**Keywords:** dairy cows, dry periods, milk, mastitis, reproduction

## Abstract

**Simple Summary:**

The goal of this study was to examine how the duration of the dry period is associated with milk production, health, and reproductive traits in a herd of cows managed with an automatic milking system. Cows with a dry period of 40–70 days showed higher energy-corrected milk production during the first 100 days of lactation compared to those with shorter or longer dry periods (*p* < 0.001). Additionally, cows in the 40–70-day dry period group exhibited the lowest milk electrical conductivity and somatic cell count, along with the highest milk lactose concentration. Mastitis pathogens were most prevalent in cows with the longest dry periods (>70 days), while *Streptococcus agalactiae* and *Staphylococcus aureus* were least detected in cows with a dry period of 40–70 days. The highest cow fertilization rate was observed in the group with the shortest dry period (<40 days). These findings provide insights that could help the dairy sector make informed decisions to enhance cow productivity and health. Moreover, managing dry period length effectively could serve as a critical tool for improving dairy herd management.

**Abstract:**

The objective of this study was to evaluate the association between dry period (DP) length and various indicators of productivity, reproduction, and udder health in cows managed with an automatic milking system. We analyzed records from 3861 cows, categorizing them into three groups based on their DP duration: (1) <40 days, (2) 40–70 days, and (3) DP > 70 days. Cows with a DP of 40–70 days had an average energy-corrected milk production that was 8.2 kg greater than that of cows with a short DP and 5.0 kg greater than that of cows with a long DP (*p* < 0.001). Milk from the 40–70-day DP group exhibited the highest lactose concentration (4.64 ± 0.01%). Additionally, cows with the longest DP had the smallest proportion of animals with a milk fat-to-protein ratio of 1.2 to 1.4. Cows with a DP of 40–70 days also showed the lowest milk electrical conductivity across all udder quarters, whereas cows with the shortest DP had the highest conductivity. The highest conception rates were observed in the group with the shortest DP. These results suggest that a DP of 40–70 days is optimal for maximizing milk production and improving both udder health and reproductive performance under AMS. Proper management of DP duration can be an effective strategy for sustainable dairy herd management.

## 1. Introduction

Research indicates that there is an optimal duration for the dry period that maximizes milk production during the following lactation [1,2,3]. While the exact optimal length can depend on factors such as breed, management practices, and individual cow characteristics, it is usually between 42 and 60 days [2]. A 55-day dry period is traditionally seen as the standard [4]. Studies have also explored the impacts of extending the dry period, and findings suggest that a period longer than this optimal length can lead to decreased milk production in the subsequent lactation [5], often due to alterations in mammary gland function and metabolic processes during an extended dry period [6].

Conversely, shortening the dry period below the recommended length can also negatively affect milk production and cow health. Church et al. [7] found that cows with a 60-day dry period produced more milk (11,942 kg) compared to those with only a 30-day dry period (10,749 kg). A shortened dry period may result in insufficient preparation of the mammary gland for the subsequent lactation, potentially causing a decrease in milk yield and an increased risk of health issues such as mastitis. Additionally, Pezeshk et al. [8] noted that shortening or eliminating the dry period could reduce or eliminate the benefits associated with milk accumulation at dry-off, which can diminish the immunodeficiency typically experienced during this period. Shortened dry periods can also disrupt the composition of mammary secretions and tissue remodeling processes, and they may affect energy balance, nutritional status, and hormonal and local regulatory mechanisms, potentially influencing the cow’s response to intramammary infections.

Beyond milk production, the duration of the dry period also impacts cow health and welfare. Longer dry periods allow cows more time to recover from the previous lactation, reduce the risk of metabolic disorders, and improve overall udder health [4]. The significance of the dry period’s duration is further underscored by the increased metabolic and infectious risks that dairy cows face during the transition from the dry period to early lactation [9,10,11].

Proper nutritional management during the dry period is crucial for influencing both milk production and cow health. Ensuring adequate nutrition, which includes a balance of energy and minerals, throughout the dry period is essential for optimizing milk production in the subsequent lactation. This is especially important due to the substantial and sudden increase in nutrient demands that occurs post-parturition to sustain milk production in dairy cattle [12,13].

The dry period is a critical phase in the dairy cow’s reproductive cycle, serving as the interval of rest between lactation cycles during which the cow ceases milk production. According to Chandler et al. [14], a dry period that extends beyond 70 days can negatively impact both milk yield and fertility, emphasizing the necessity of optimal management practices to ensure successful lactation cycles.

The optimal dry period is important for maximizing not only the quantity of milk produced by dairy cows but also for improving its quality and composition [15,16]. The duration of the dry period influences various aspects of milk composition, such as fat, protein, and lactose content. Research indicates that changes in the length of the dry period can affect milk composition, though these effects are influenced by factors including nutritional management during the dry period and the genetic traits of the cows [17,18,19].

Moreover, the duration of the dry period is important for ensuring mammary health. Extended dry periods can increase the risk of mastitis, a common udder infection in dairy cows, whereas excessively short dry periods may prevent the udder from fully recovering, leading to a higher risk of mastitis and other udder-related issues [20,21,22,23].

In addition to its effects on mammary health, the length of the dry period has significant implications for reproductive performance. Both very short and excessively long dry periods can adversely affect a cow’s ability to conceive and maintain a pregnancy, thereby impacting the herd’s overall fertility and economic performance [24,25].

A comprehensive study on Italian dairy farms, which reviewed 48,297 lactation records from multiparous cows, revealed that dry periods of 61 to 70 days and 50 to 60 days were associated with the highest 305-day milk production, whereas dry periods shorter than 40 days were linked to the lowest milk production. Furthermore, dry periods of less than 40 days and more than 70 days were associated with significantly higher odds of culling within the first 60 days of lactation compared to the 50–60 days dry period group. The study also found that the likelihood of successful fertilization within the first 200 days of lactation was highest with dry periods of 40 to 49 days and 50 to 60 days, while dry periods of less than 40 days, 61 to 70 days, and greater than 70 days were associated with lower fertility [26].

In recent years, the dairy industry has experienced consistent growth in herd size, coupled with a reduction in the labor force. This has driven the adoption of automation and precision animal husbandry technologies to maintain productivity and efficiency [27,28]. A notable technological advancement in the dairy sector has been the implementation of automatic milking systems (AMS) [29,30]. AMS represent a significant leap forward in enhancing both the health and production efficiency of dairy herds [31], allowing farmers to shift their focus from managing milking routines to overall herd management [32].

AMS generate extensive data for each cow, including critical milk characteristics such as somatic cell count and electrical conductivity, which are essential for detecting mastitis. The substantial data volumes produced by AMS, compared to traditional productivity control systems, facilitate precise and real-time monitoring of herd productivity and health indicators at the individual animal level [33,34].

To date, there is a notable lack of comprehensive data analyzing the relationship between dry period length and AMS indicators. The scientific literature highlights the importance of optimal dry period management for improving milk production, cow health, and reproductive performance. Integrating AMS data into this analysis could provide deeper insights into the multifaceted impacts of dry period management.

The objective of this study was to examine the association between dry period length and various indicators of productivity, reproduction, and udder health in a herd of cows using an automatic milking system. The findings from this study aim to inform dairy farmers and industry stakeholders about best practices for balancing productivity and health, ultimately contributing to more sustainable herd management.

## 2. Materials and Methods

### 2.1. Study Design and Animals

This study was conducted in compliance with the Law on Animal Welfare and Protection of the Republic of Lithuania (study approval number PK016965) on a commercial dairy farm located in the central region of Lithuania. The research spanned from January 2021 to January 2024, and included a dataset of 3862 records from 2003 cows.

The dry period was documented for cows after their first, second, and third lactations, and productivity indicators were analyzed for the second to fourth lactations within the first 100 days of each lactation. The cows selected for the study were healthy, with no clinical symptoms of disease, and evaluations began from at least the 200th day of pregnancy. Their body condition scores were assessed on a five-point scale, developed by Ferguson et al. [35], on the 200th day before calving, at pre-calving, and on the 100th day of lactation.

The cows were housed in a barn year-round under a zero-grazing system and were milked using Lely Astronaut^®^ A3 milking robots with a free traffic system. The total mixed ration (TMR) provided was formulated to meet the energy and nutritional requirements of 550–650 kg Holstein cows (see Table 1).

The cows underwent mastitis testing, which included a bacteriological test for mastitis pathogens and an antibiotic sensitivity test performed by JSC “Pieno Tyrimai” Kaunas, Lithuania.

Treatment was carried out according to established protocols. Testing was triggered by an increase in somatic cell count (>200,000 cells/mL), an electrical conductivity value above 6.5 mS/cm, an electrical conductivity difference between udder quarters greater than 1 mS/cm, or the presence of visual signs of mastitis [36].

### 2.2. Study Data

The final dataset was refined to exclude records from cows (*n* = 351) that had calving ease scores above 3, calves that died within 24 hours of birth, cases of twin births, and cows with a gestation period of less than 260 days. The remaining data were classified into three dry period length categories: (1) <40 days, (2) 40–70 days, and (3) >70 days.

Data from the automated milking systems included daily measurements of milk yield, fat content, protein content, lactose content, somatic cell count, and electrical conductivity of udder quarters, recorded for the initial 100 days of lactation. In addition, insemination outcomes were documented, focusing on the number of cows fertilized after the first insemination attempt and the percentage of cows achieving fertilization within the first 100 days of lactation, categorized by dry period length.

The milk yield was adjusted to energy-corrected milk (ECM) using the following equation [37]:

ECM = 0.327 × Milk Yield (kg) + 12.95 × Fat (kg) + 7.65 × Protein (kg)

Cows were further categorized into three subgroups based on their average milk fat-to-protein ratio (F/P): (1) F/P < 1.2, (2) 1.2 ≤ F/P ≤ 1.4, and (3) F/P > 1.4. The F/P ratio < 1.4 was used as a marker for subclinical acidosis [38], whereas a ratio > 1.2 was indicative of potential subclinical ketosis [39]. The cows did not present clinical signs of these diseases.

### 2.3. Statistical Analysis of Data

The study’s outcome variables comprised milk yield, fat percentage, protein percentage, lactose percentage, somatic cell count, electrical conductivity of udder quarters, health and fertility events, and mastitis incidence, with dry period length as the explanatory variable. Data were averaged for the first 100 days of lactation for each cow before analysis.

Statistical analysis was conducted with SPSS 25.0 software (IBM Corp. Released 2017. IBM SPSS Statistics for Windows, Version 25.0. Armonk, NY: IBM Corp., Armonk, NY, USA). Statistical characteristics such as sample size (n), mean (M), and standard error of the mean (SEM) were computed. The Shapiro–Wilk test was performed to check data normality, and the Bonferroni post hoc test was used for comparing differences among the three dry period length groups. Pearson’s chi-square test was employed to assess differences in frequencies across subgroups based on fat-to-protein ratio (F/P), level of milk lactose, somatic cell count and electrical conductivity, mastitis pathogens, and fertilization status.

A probability of less than 0.05 was considered statistically significant (*p* < 0.05) for all parametric and non-parametric tests.

## 3. Results

The analysis showed that the average dry period length of cows was 59.3 ± 0.29 days. The first group comprised 25.3% of the total, the second group made up 52.2%, and the third group represented 22.5% of cows. The average dry period for cows in the first group was 33.2 ± 0.39 days, in the second group it was 62.7 ± 0.87 days, and in the third group it was 80.7 ± 1.21 days.

### 3.1. Milk Characteristics in Cows Based on Their Dry Period Length

The analysis showed that cows with a dry period lasting 40–70 days had the highest average daily milk yield, while those with a dry period of <40 days had the lowest. The highest average milk fat content was also found in cows with a dry period of 40–70 days, and the highest milk protein content was observed in cows with a dry period of >70 days. The lowest milk fat and protein percentages were observed in the first group of cows with the shortest dry period. In the milk of the second group of cows, the highest concentration of milk lactose was also found (4.64 ± 0.01%)—0.2% higher than cows with a long dry period and 0.4% higher than cows with a short dry period (*p* < 0.05). Cows with an average lactose content above 4.60% accounted for 40.2% of all animals in the second group, 36.2% in the first, and 31.9% in the third (Table 2).

**Figure 1:**
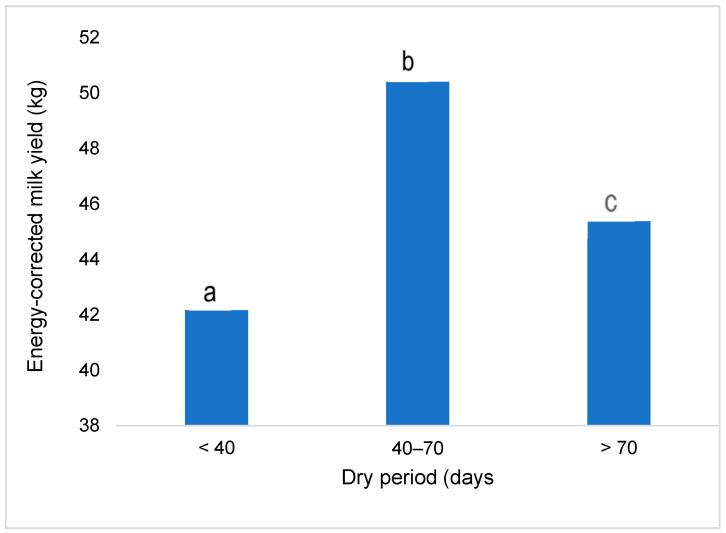
Energy-corrected milk yield (kg) in cows categorized by dry period length. The difference in mean values between groups is statistically significant (*p* < 0.05) when marked with different letters.

Taking into account the average daily milk yield of cows considering their milk fat and protein content (Figure 1), the quantity of energy-corrected milk for cows with a dry period of 40–70 days was 8.2 kg higher than for cows with a short dry period and 5.0 kg higher than for cows with a long dry period (*p* < 0.001).

The lowest number of cows with a milk fat-to-protein ratio between 1.2 and 1.4 was found in the group with the longest dry period (4.3 percentage points lower than the group with a dry period of 40–70 days and 10.6 percentage points lower than the group with the shortest dry period, *p* < 0.05).

**Figure 2:**
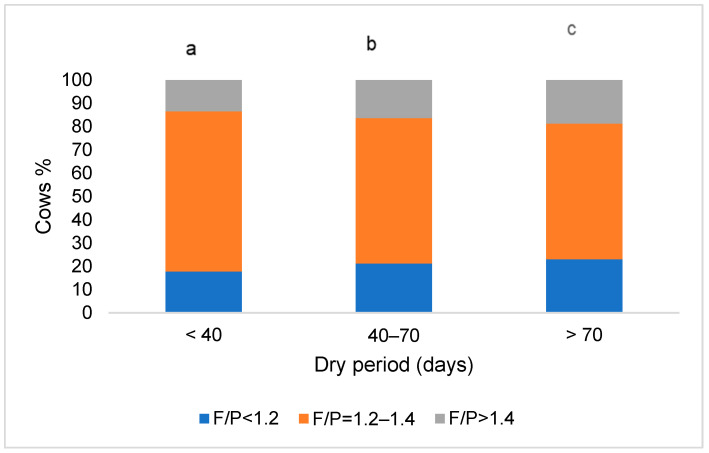
Distribution of cows based on their dry period length and milk fat-to-protein ratio. The difference in mean values between groups is statistically significant (*p* < 0.05) when marked with different letters.

The highest number of animals with a milk fat-to-protein ratio > 1.4 was found in the group of cows with the longest dry period (2.1–6.7 percentage points more animals compared to other groups, *p* < 0.05), as well as cows with a milk fat-to-protein ratio < 1.2 (2.4–5.3 percentage points more, *p* < 0.05). A graphical summary of the data on the distribution of cows based on their dry period length and milk fat-to-protein ratio is presented in Figure 2.

**Figure 3:**
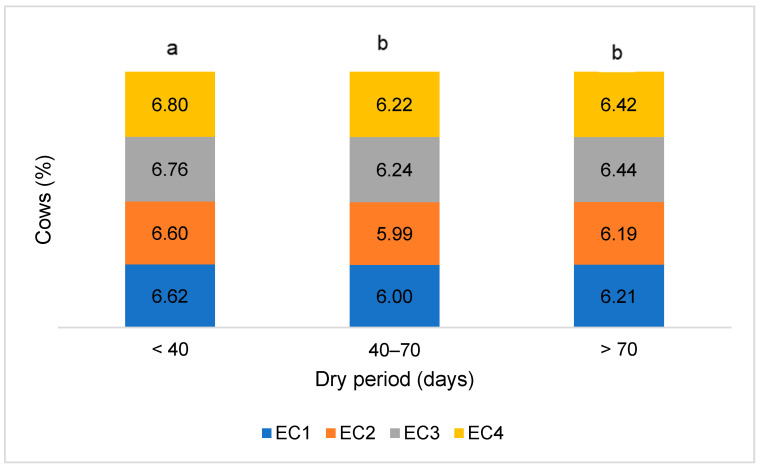
Milk electrical conductivity (mS/cm) in cows categorized by dry period length. EC1—front left udder quarter, EC2—front right udder quarter, EC3—rear left udder quarter, EC4—rear right udder quarter. The difference in mean values between groups is statistically significant (*p* < 0.05) when marked with different letters.

The lowest milk electrical conductivity in all udder quarters was observed in cows with a dry period of 40–70 days, while the highest was in cows with the shortest dry period (Figure 3). The milk electrical conductivity of all groups of cows in the rear udder quarters was higher than in the front ones.

**Figure 4:**
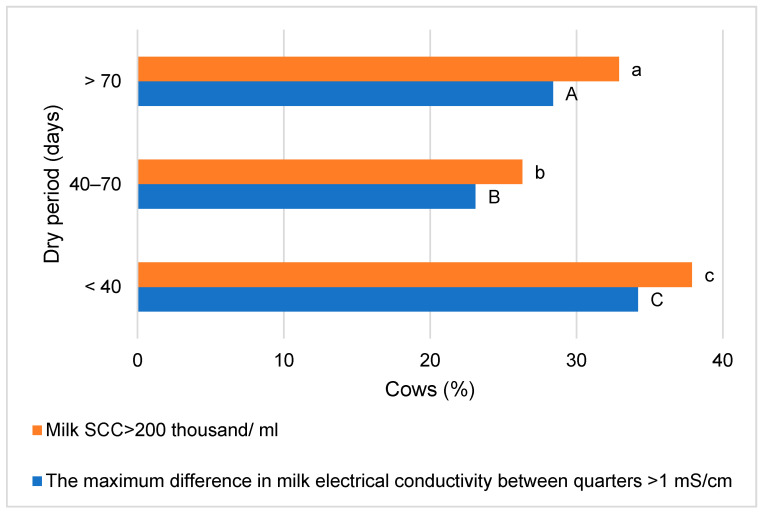
Distribution of cows by milk somatic cell (SCC) level and difference in milk electrical conductivity between udder quarters. The difference in mean values between groups is statistically significant (*p* < 0.05) when marked with different letters: a, b, c (for milk somatic cell) or A, B, C (for maximum difference in milk electrical conductivity between udder quarters).

In the group of cows with the shortest dry period, the highest number of cows with milk somatic cell levels > 200 thousand/mL was observed, as well as those with a maximum difference in milk electrical conductivity between udder quarters > 1 mS/cm (11.6 and 5.8 percentage points less, respectively, than in the second group of cows, where the averages of these indicators were the lowest; *p* < 0.05). The data mentioned are presented in Figure 4.

### 3.2. Prevalence of Mastitis Pathogens in Association with Dry Period Length in Cows

**Figure 5:**
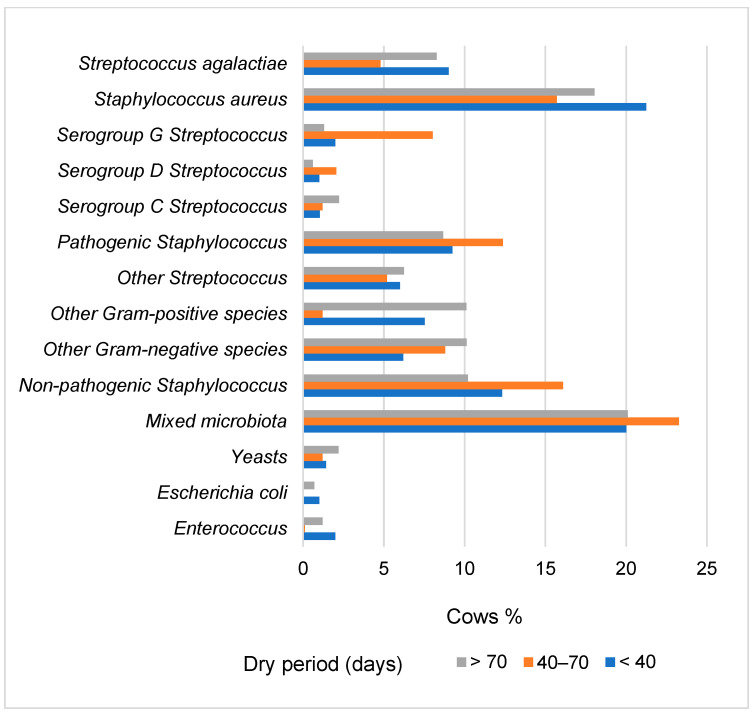
Prevalence of the mastitis agents in milk samples categorized by dry period length of cows.

After microbiological testing, mastitis pathogens were identified in samples from 25.1% of cows with a dry period of 40–70 days, 32.2% of cows with the shortest dry period, and 35.3% of cows with the longest dry period (Figure 5).

In the group of cows with a dry period of 40–70 days, microbiological tests showed the highest frequency of Serogroup G *Streptococcus* (6.02–6.72 percentage points higher, *p* < 0.001), non-pathogenic *Staphylococcus* (3.79–5.90 percentage points higher, *p* < 0.05), pathogenic *Staphylococcus* (3.11–3.69 percentage points higher, *p* < 0.05), mixed microbiota (3.17–3.27 percentage points higher, *p* < 0.05), and serogroup D *Streptococcus* (1.06–1.46 percentage points higher) compared to other groups (Figure 4). The lowest number of samples with *Streptococcus agalactiae* (4.8%) and *Staphylococcus aureus* (15.7%) pathogens was found in the group of cows with a dry period of 40–70 days, which was 1.35 to 1.88 times lower (*p* < 0.05) than in cows with the shortest and longest dry periods. In the group of cows with the longest dry period, the highest prevalence of other Gram-negative species (1.32–3.93 percentage points higher compared to other groups) and Gram-positive species (2.58–8.91 percentage points higher, *p* < 0.05) was observed (Figure 5).

### 3.3. Association of Body Condition Score and Fertility Characteristics with Dry Period Length in Cows

The average body condition score of cows on the 200th day before calving was 3.52 ± 0.14. A similar, but slightly lower, average score of 3.47 ± 0.12 was recorded at pre-calving. On the 100th day of lactation, the average body condition score (3.08 ± 0.14) was significantly lower than the pre-calving score (*p* < 0.05).

**Figure 6:**
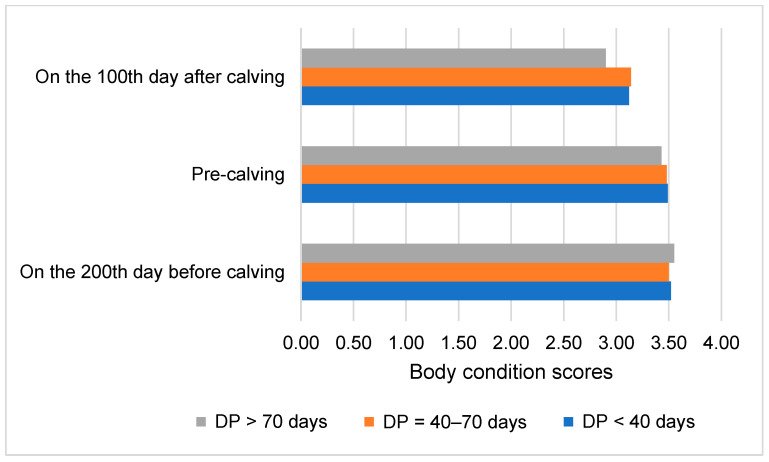
Body condition scores of cows based on dry period length. DP—dry period. Group 1: DP < 40 days, Group 2: DP = 40—70 days, Group 3: DP > 70 days.

There were no significant differences in body condition scores among the cow groups across the assessed periods. However, there was a significant decrease in body condition scores from pre-calving to the 100th day of lactation, with a reduction of 0.34 in Group 2 and 0.53 in Group 3 (*p* < 0.05). This finding suggests that a longer dry period is associated with a greater loss in body condition score by the peak of lactation (Figure 6).

**Figure 7:**
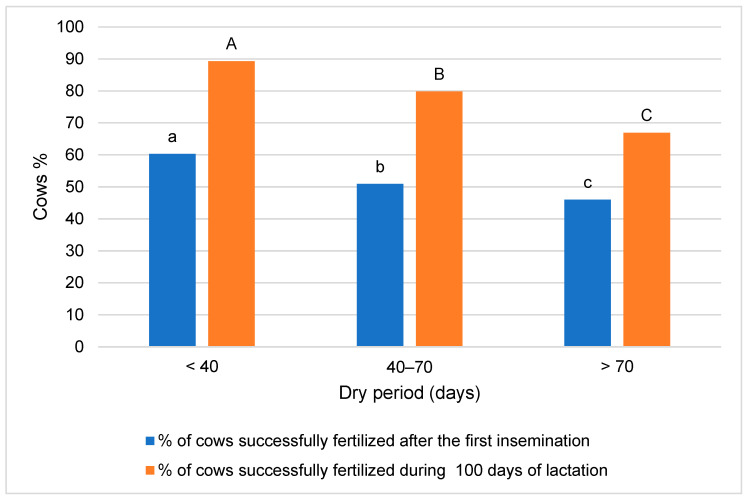
Cow insemination and conception results considering their dry period length. The difference in mean values between groups is statistically significant (*p* < 0.05) when marked with different letters: a, b, c (for % of cows successfully fertilized after the first insemination) or A, B, C (for % of cows successfully fertilized during 100 days of lactation).

As shown in the analysis data presented in Figure 7, the highest number of cows was successfully fertilized in the shortest dry period group, both after the first insemination (9.4–14.3 percentage points higher compared to other groups, *p* < 0.001) and after 100 days of lactation (9.5–22.4 percentage points higher, *p* < 0.001), while the lowest number was in the longest dry period group.

## 4. Discussion

The dry period is essential for renewing aging mammary epithelial cells and enhancing udder tissue before the subsequent lactation [2]. During World War II, the UK implemented the 305-day lactation and 60-day dry period regimen to maximize milk production and accelerate genetic progress due to food shortages [40].

As of 2022, the average dry period length in the United States has increased to approximately 60.8 days, with over 72% of dairies adopting a dry period of 60 days or longer. This trend reflects a sustained practice in the industry aimed at optimizing both milk production and cow health [40]. Van Knegsel et al. [41] found that shortening or omitting the dry period led to reduced total milk yield, lower lactose percentage, and decreased yields of lactose, fat, and protein during the first 14 weeks of lactation (*p* < 0.05).

In the three weeks before calving, fetal nutritional demands peak, yet dry matter intake typically decreases by about a third [42]. Early lactation energy requirements for milk production increase rapidly, often exceeding energy intake [1,42]. As a result, transition cows frequently experience negative energy balance (EB), leading to the mobilization of body reserves (reduced body condition score) and associated metabolic disorders such as ketosis, mastitis, and reduced fertility [43,44].

Automatic milking systems equip farmers with comprehensive datasets and metrics crucial for effective management of livestock productivity and health, as well as for early predictive analytics [29,30,32,34]. O‘Callaghan and colleagues [44] suggest that milk lactose concentration and electrical conductivity are among the most effective parameters for monitoring and detecting subclinical and clinical mastitis, particularly when combined with milk somatic cell count. Recent studies have identified an individual milk electrical conductivity greater than 6.5 mS/cm and a difference of more than 1.0 mS/cm between the udder quarters with the highest and lowest conductivity as diagnostic thresholds for mastitis infection [36].

Our study data revealed that cows with a dry period of 40–70 days had the lowest milk somatic cell count and milk electrical conductivity, as well as the highest milk lactose concentration. This group also showed the lowest number of cows with a maximum difference in milk electrical conductivity between udder quarters > 1 mS/cm and the lowest prevalence of mastitis pathogens. Conversely, the highest prevalence of mastitis pathogens (35.3%) was observed in milk samples from cows with the longest dry period.

The incidence of mammary gland infections is highly problematic within the dairy industry, leading to production losses and poor cow performance. Both Gram-negative and Gram-positive organisms can produce endotoxins that contribute to mammary gland infections and activate the immune system [45]. To our knowledge, there is still a lack of research on the relationship between dry period length and the prevalence of different mastitis pathogens during lactation.

The analysis revealed differences in the prevalence of various mastitis pathogens among cow groups based on dry period length. The highest prevalence in milk samples was of mixed microbiota (20.0–23.3%) and *Staphylococcus aureus* (15.7–21.3%). The lowest frequency of *Staphylococcus aureus* and *Streptococcus agalactiae* pathogens was found in the group of cows with a dry period of 40–70 days (Figure 5). The primary pathogen associated with contagious mastitis is *Staphylococcus aureus*, known to be among the most challenging pathogens to manage and treat [46].

Cows experiencing prolonged dry periods tend to accumulate more fat, which can predispose them to metabolic challenges. Furthermore, Santschi et al. [47] reported that cows with dry periods longer than two months were at a higher risk of developing ketosis (as confirmed by our research data presented in Figure 2), potentially impacting fertility, especially the conception rate after the first service.

Decreased reproductive efficiency in dairy herds is an important economic concern. Pinedo et al. [48] observed that cows with a dry period exceeding 77 days experienced a longer time until first insemination and a prolonged interval from calving to conception, and needed more services per conception compared to cows with a standard dry period.

Cow productivity and fertility indicators are closely related to body condition score (BCS), which measures body fat in dairy cattle. BCS is a widely accepted and practical method for assessing changes in energy reserves and is a crucial factor in dairy cattle management [49,50]. However, there is still a lack of knowledge about how BCS changes in relation to the length of the dry period in cows.

Prolonged dry periods (>70 days) were associated with a greater loss in BCS at the peak of lactation, as shown in Figure 6 of our study. A significant loss in BCS can lead to decreased fertility, as cows with lower BCS are less likely to conceive successfully.

This study found that the lowest success in cow fertilization (Figure 6) occurred in the group with the longest dry period, both after the first insemination and after 100 days of lactation. Cows with extended dry periods were at a higher risk of developing metabolic disorders, which can compromise fertility. These conditions can disrupt the hormonal balance necessary for successful ovulation and conception.

In the group of cows with the shortest dry period, we found the highest number of cows with milk somatic cell levels >200 thousand/ml and the worst productivity. While a longer dry period can potentially allow for more complete mammary gland involution and recovery, it also poses significant risks to fertility and overall cow health. Optimal dry period management is crucial for balancing these factors to ensure both high milk production and good reproductive performance [5,26].

## 5. Conclusions

Cows with a dry period of 40–70 days demonstrated the highest energy-corrected milk yield compared to those with shorter or longer dry periods, indicating that a 40–70-day dry period is optimal for maximizing milk production. The longest dry period was associated with the highest proportion of cows exhibiting a milk fat-to-protein ratio > 1.4 and <1.2, suggesting potential metabolic challenges such as ketosis or acidosis.

Cows with a dry period of 40–70 days also had the lowest milk electrical conductivity and somatic cell count, indicating better udder health. Additionally, this group showed the lowest prevalence of mastitis pathogens, including fewer cases of *Streptococcus agalactiae* and *Staphylococcus aureus*. The highest cow fertilization rates were observed in the group with the shortest dry period, while the lowest rates were found in the group with the longest dry period, highlighting that a longer dry period is negatively associated with reproductive performance.

Overall, a dry period length of 40–70 days is associated with optimal outcomes for milk production, milk composition, udder health, and reproductive performance. Conversely, a dry period exceeding 70 days is linked to reduced milk production and lower fertility rates, making it less favorable for dairy herd management.

## Figures and Tables

**Table 1:** Composition of the total mixed ration for dry cows.

Component	Amount (kg/cow/day)
Rapeseed 36%	1.2
Grass silage (27% DM)	8
Maize silage (27% DM)	1.2
Wheat straws	7.5
Water	4.3
Dry cow mineral and vitamin	0.250

DM—Dry matter.

**Table 2:** Milk productivity and content traits in cows by dry period length.

Group of Cows	n	Dry Period Length (Days)	Milk Yield, kg	Milk Fat %	Milk Protein %	Milk Lactose %
1	977	<40	38.21 ^a^ ± 0.27	4.01 ^a^ ± 0.02	3.36 ^a^ ± 0.01	4.60 ^a^ ± 0.01
2	2015	40–70	44.81 ^b^ ± 0.21	4.14 ^b^ ± 0.01	3.42 ^b^ ± 0.01	4.64 ^b^ ± 0.01
3	870	>70	40.77 ^c^ ± 0.16	4.03 ^a^ ± 0.02	3.45 ^b^ ± 0.01	4.62 ^ab^ ± 0.01

The difference in mean values between groups is statistically significant (*p* < 0.05) when marked with different letters.

## Data Availability

The data presented in this study are available within the article.
